# Animal Models of Peritoneal Dialysis: Thirty Years of Our Own Experience

**DOI:** 10.1155/2015/261813

**Published:** 2015-07-06

**Authors:** Krzysztof Pawlaczyk, Ewa Baum, Krzysztof Schwermer, Krzysztof Hoppe, Bengt Lindholm, Andrzej Breborowicz

**Affiliations:** ^1^Department of Nephrology, Transplantology, and Internal Medicine, Poznan University of Medical Sciences, Poznan, Poland; ^2^Divisions of Renal Medicine and Baxter Novum, Department of Clinical Science, Intervention, and Technology, Karolinska Institute, Stockholm, Sweden; ^3^Department of Pathophysiology, Poznan University of Medical Sciences, Ulica Rokietnicka 8, 60-806 Poznan, Poland; ^4^Higher Vocational State School, Kalisz, Poland

## Abstract

Experimental animal models improve our understanding of technical problems in peritoneal dialysis PD, and such studies contribute to solving crucial clinical problems. We established an acute and chronic PD model in nonuremic and uremic rats. We observed that kinetics of PD in rats change as the animals are aging, and this effect is due not only to an increasing peritoneal surface area, but also to changes in the permeability of the peritoneum. Changes of the peritoneal permeability seen during chronic PD in rats are comparable to results obtained in humans treated with PD. Effluent dialysate can be drained repeatedly to measure concentration of various bioactive molecules and to correlate the results with the peritoneal permeability. Additionally we can study in *in vitro* conditions properties of the effluent dialysate on cultured peritoneal mesothelial cells or fibroblasts. We can evaluate acute and chronic effect of various additives to the dialysis fluid on function and permeability of the peritoneum. Results from such study are even more relevant to the clinical scenario when experiments are performed in uremic rats. Our experimental animal PD model not only helps to understand the pathophysiology of PD but also can be used for testing biocompatibility of new PD fluids.

## 1. Introduction

An important step in studying various aspects of peritoneal dialysis (PD) is to establish an animal model which can mimic a clinical situation and can be reproduced. Different animal models of peritoneal dialysis have been used in recent years [1–20]. Experimental animal models of peritoneal dialysis have been used both to study the physiology of peritoneal transport [21, 22] and for testing biocompatibility of dialysis solutions [23, 24]. Most of these studies were performed on nonuremic animals. The transgenic mouse and cellular models become available to target other relevant pathways and, with the application of multiplex assay and DNA/RNA array technologies in these models, it will become possible to assess the interactive relationships of various physiological and pathophysiological pathways in the peritoneum in relation to the systemic parameters [18].

In our lab we established an acute and chronic PD model in nonuremic rats and mouse [3, 19, 20, 23, 25–36]. This model has been modified to evaluate the different aspects of peritoneal dialysis [3, 19, 20, 37–48]. The main objective of our research was to evaluate the usefulness of the peritoneal dialysis animal model as a means to evaluate the changes taking place during the treatment with peritoneal dialysis and correlate these experimental results with those in a clinical setting. All the experiments were performed according to protocol approved by Animal Ethics Committee of the authors' institution.

## 2. Topography of the Peritoneal Cavity

When we compared the contribution of different parts of the peritoneum to the total peritoneal surface area in humans, rabbits, and rats, significant differences were found [49]. The area of the diaphragm, which seems to play an important role in lymphatic drainage from the peritoneal cavity [50], is relatively larger in humans than in experimental animals [49]. Therefore, one may speculate that data from experimental studies evaluating the rate of the lymphatic drainage of dialysate performed in rats or rabbits may underestimate the significance of that process in humans [49]. The transperitoneal transport of water and solutes depends on the effective peritoneal surface area, which reflects the density of microvessels in the membrane, and is related to the anatomical area of the peritoneal membrane [34, 49]. Since the parietal peritoneum is larger in rats than in humans, one may speculate that results from the animal studies may overestimate the significance of that effect when compared to humans [34, 49]. We observed that kinetics of peritoneal dialysis in rats change as the animals age, and this effect is due not only to an increasing peritoneal surface area, but also to changes in the permeability of the peritoneum [34]. Thus, we should take into consideration these variations when comparing the results from* in vivo *experiments performed on rats of various ages and weights [34].

## 3. Animal Models of Acute Peritoneal Dialysis for Study of the Physiology of the Transperitoneal Transport of Solutes

During peritoneal dialysis there is a continuous exchange of fluids and solutes between the blood and dialysis fluid dwelling in the abdominal cavity. Due to hypertonicity of the dialysis solution, water is removed from the bloodstream into the peritoneal cavity and at the same time there is a bidirectional transport of solutes: glucose is absorbed into the blood and metabolites are diffusing from blood into the dialysate which results in cleaning of the body from the toxic compounds. Additionally, water removal during the process of peritoneal dialysis depends also on the amount of fluid which is drained from the peritoneal cavity by lymphatics [51].

In our lab we used an acute model of peritoneal dialysis which was initially performed in rabbits and afterwards in rats. In rats under short ether anesthesia, the abdominal cavity was punctured and dialysis solution was infused intraperitoneally. After a few minutes animals were awoken, with free access to food and water. At designated time periods, a group of animals was sacrificed by means of an anesthesia overdose. The abdominal cavity was opened and the residual dialysate was collected for measurements. Simultaneously blood samples were obtained from the heart. Using such a relatively simple model of dialysis, we were able to describe various mechanisms of water and solute transport during peritoneal dialysis. We found that alkalization of the dialysis fluid enhances lactate removal in animals with lactic acidosis due to hypoxia [52]. In another study, the enhancing effect of the local anesthetic procaine on peritoneal transport of solutes was reported and additionally, using our* in vitro* model of the isolated peritoneal membrane, we confirmed that procaine had a direct effect on mesothelial permeability [53]. In subsequent experiments combining* in vivo* acute model of peritoneal dialysis in rabbits and* in vitro* study on the isolated peritoneal membrane, we also found that the effect of bupivacaine on the transperitoneal transport of solutes during dialysis was due to its direct action on the mesothelial cells [54, 55]. Using combined* in vivo* and* in vitro* experiments we documented that reduced peritoneal permeability to water and solutes in presence of chondroitin sulphate is due to its action on the peritoneal interstitium [36]. During acute experiments on rats we showed that inhibition of the intraperitoneal synthesis of nitric oxide resulted in an increased selectivity of the peritoneal permeability and an increased net ultrafiltration [32]. In another series of studies we investigated inflammatory states and changes in peritoneal transport of water and other molecules during acute peritoneal dialysis in rats after lipopolysaccharide (LPS) application [39]. The addition of LPS to a standard glucose-based dialysis solution induces a strong and acute intraperitoneal inflammatory reaction reflected by increased dialysate cell count, increased cytokine and VEGF levels, as well as increased solute transport, and decreased ultrafiltration, in a dose-dependent manner [39]. The changes in peritoneal transport in this* in vivo* rat model of acute LPS-induced peritoneal inflammation are similar to results obtained in continuous ambulatory PD patients in the early phases of the peritonitis [39]. Our finding that increased VEGF levels correspond to the intensity of inflammation supports the hypothesis that inflammation could be a key component of VEGF stimulation [39].

The last group of our experiments on the model of the acute peritoneal dialysis in rabbits focused on evaluation of factors affecting lymphatic drainage from the peritoneal cavity filled with the dialysis solution. We found that peritoneal lymphatic drainage is not steady during the intraperitoneal dialysate dwell and its value is modified by factors such as volume of the dialysate, its tonicity, and presence of protein or uremia [56].

In conclusion, we think that despite their technical simplicity, acute peritoneal dialysis experiments, especially when combined with* in vitro* studies on the isolated peritoneal membrane, provide vast information about function of the peritoneum as the dialysis membrane.

## 4. Biocompatibility of Peritoneal Dialysis

Peritoneal dialysis is not a biocompatible procedure. Intraperitoneal infusion of any solution results in initiation of an inflammatory reaction, causing progressive injury to the peritoneum. Additionally, dialysis fluid has nonphysiological composition (i.e., low pH, hypertonicity, and high-glucose concentration) which on one hand stimulates an inflammatory reaction and on the other hand may have a direct injuring effect to the peritoneum [57]. Some of the results found in experimental “short-term studies,” lasting several hours, may not be the same as those observed in patients maintained on PD for a long time [23, 37, 47, 48, 58, 59]. We developed an experimental chronic peritoneal dialysis model for repeated dwell study in rats. Application of the model of chronic peritoneal dialysis in rats where animals are exposed to the tested solutions for at least 4 weeks allows evaluation of their effects on structure and function of the peritoneum [48].

### 4.1. Catheter Implantation: Surgical Procedure (Figures 1 and 2)

**Figure 1:**
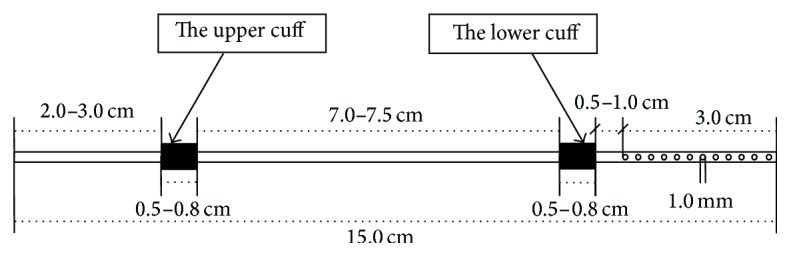
The structure of the catheter.

**Figure 2:**
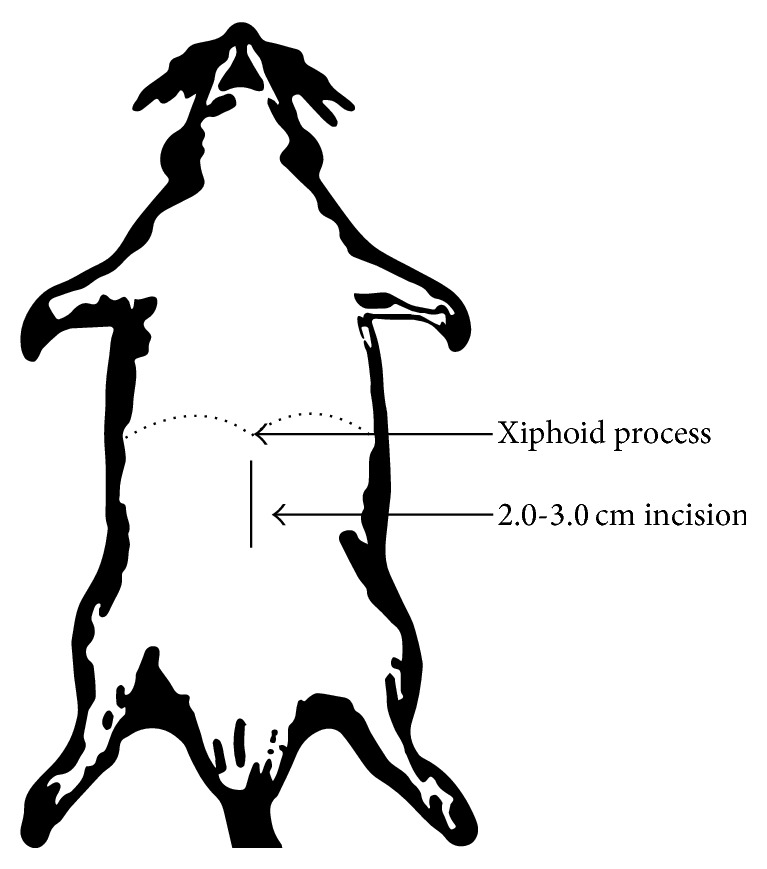
The site of incision.

The catheters, patterned after a standard Tenckhoff catheter, were constructed from medical silicone tubing with two polyester cuffs (Figure 1). The peritoneal catheter was implanted in rats according to a described method [3, 23, 24, 35, 43]. Under anesthesia (Medetomidine and Midazolam, i.m. or ether inhalation), first a 3 cm long incision of skin on the abdomen was performed and the abdominal muscles (about 2 cm long incision) were cut (Figure 2). A vertical incision was made in the mid-line (in order to avoid bleeding) beneath the xiphoid process (Figure 2). Then the peritoneal cavity was opened and omentectomy was performed. The distal part of the sterile peritoneal catheter was inserted into the abdominal cavity. The cuff was attached to the superficial muscle layer by purse string sutures. The catheter was pulled up through a subcutaneous track up to the animal's neck and exteriorized between the ears [23, 37, 47, 48, 58, 59]. Then 10–15 mL of peritoneal dialysis fluid was infused into the peritoneal cavity via the catheter and immediately drained. After recovering from the surgical procedures, animals returned to their cages and were allowed full mobility over the period of the experiment. After implantation of the catheters, animals were randomly divided into experimental groups.

### 4.2. Dialysis Procedure

During the first week after catheter implantation, the instilled volume of standard glucose-based solution with antibiotics (Netilmicin 5 mg/L and Cefuroxime 60 mg/L) was gradually increased from 10 to 20 mL. Tested solutions were infused daily (1–4 times) into the peritoneal cavity via the catheter. The instilled fluid was allowed to absorb gradually from the abdominal cavity or drained after the completed dwell time. During the study, the animals were awake for infusion and drainage of fluids.

### 4.3. PET (Peritoneal Equilibration Test)

PET was performed during a 2- or 4-hour exchange with hypertonic peritoneal dialysis solution according to the protocol used in our lab [27, 30, 37, 48]. Under ether anesthesia, a blood sample was drawn from the tail vein in each rat. Then, 30 mL of dialysis solution was infused into the peritoneal cavity of the animal. During the 4-hour exchange, the animals were awake, with free access to water and food. Dialysate samples were drawn at time 0 (instantly after infusion of the dialysate) at 30 minutes, 1 hour, 2 hours, and 4 hours of the dwell. After four hours, the residual dialysate in each rat was drained and its volume measured. Peritoneal permeability to glucose was assessed based on the decline of glucose concentration in the dialysate expressed by the ratio D/D_0_ (D, glucose concentration in the dialysate sample; D_0_, glucose concentration in the dialysate at time 0). Peritoneal permeability to other solutes was measured by calculating the ratio of their concentration in the dialysate sample to their concentration in plasma.

Changes of peritoneal permeability seen during* in vivo* experimental models of chronic peritoneal dialysis in rats are comparable to results obtained in humans on CAPD [27, 60]. For the first time, we described an experimental chronic peritoneal dialysis model with repeated dwell studies with drainage in nonuremic rats and evaluated the effects of addition of heparin to glucose-based peritoneal dialysis fluid on peritoneal fluid and solute transport [3]. Heparin may improve peritoneal fluid transport possibly as a result of better healing and reduced peritoneal inflammation, as was shown in this novel animal model of chronic peritoneal dialysis with repeated dwell studies [3]. We consider that repeated dwell studies with drainage might improve the understanding of changes in transperitoneal permeability during peritoneal dialysis [3, 27, 60]. The advantage of this model in relation to other peritoneal dialysis animal models is the ability to conduct peritoneal dialysis with fluid exchanges where the fluid drainage is conducive to effluent cell analysis and active substance concentration measurements [3, 27, 30, 37, 38, 40, 58]. It is of importance to evaluate, in a continuous fashion, transport across the membrane during the study and perform histopathological tissue assessment at the end of the experiment [38, 40, 42].

In studies using our experimental model of chronic peritoneal dialysis in rats we found that glucose has a more injurious effect on the peritoneum in comparison to mannitol [46]. At the same time hypertonic dialysis solutions containing glucose are less injurious than phosphate-buffered saline (PBS) fluid [26, 47]. In another study we found that dialysis fluids with neutral pH and low concentration of glucose degradation products cause a weaker intraperitoneal inflammatory reaction and fibrosis of the peritoneum, as opposed to the standard acidic solutions [25]. Using our model we were able to study not only the effects of chronic peritoneal dialysis on structure and function of the peritoneum, but also the function of the peritoneal cells responsible for the local host defense against infections. Hypertonicity of the dialysis solutions suppresses the function of the peritoneal cells, which may result in less injury to the peritoneum, yet at the same time increases predisposition to intraperitoneal infections [28]. On the other hand, new generations of the dialysis solutions with neutral pH and low concentration of GDPs suppress intraperitoneal inflammation while the function of the peritoneal leukocytes seems to improve, as reflected by a stronger response to endotoxin [30].

In another series of studies using our experimental model we looked at potential approaches which may result in better biocompatibility of the dialysis solutions. We did not find any advantage of the dipeptide glycylglycine as an alternative to glucose osmotic solute [33], but we did a whole series of studies which confirmed that N-acetylglucosamine (NAG) is safer and more biocompatible than glucose osmotic solute, suggesting that NAG can potentially be used in dialysis solutions [35]. We found also that NAG could be a better osmotic solute than glucose as it, in addition, does not cause systemic hyperinsulinemia [44]. Hypertonic solutions containing NAG have less suppressive effects than glucose on function of the peritoneal leukocytes [31] but at the same time suppress the intraperitoneal inflammation during acute peritonitis [41]. We found in* in vitro* experiments that NAG stimulates hyaluronan synthesis in peritoneal mesothelial cells and fibroblasts [61] which was confirmed in rats chronically exposed to the dialysis solution containing NAG as an osmotic solute [62]. In these animals there was an increased amount of hyaluronan in the peritoneal interstitium which resulted in reduced transperitoneal loss of proteins and increased net UF due to slower absorption of dialysate from the abdominal cavity. Our findings regarding NAG confirm our previous results from experiments with hyaluronan in the model of chronic dialysis in rats. In animals exposed to chronic peritoneal dialysis with the standard solution but supplemented with hyaluronan, less intraperitoneal inflammation and less peritoneal fibrosis were observed and, at the same time, transperitoneal protein loss was reduced whereas net UF increased [29].

This chronic peritoneal dialysis rat model allows us to examine also the influence of different substances exemplified by GDPs on changes in renal function and kidney structure in a case of reduced number of nephrons [38] and to achieve a background very similar to the clinical setting in patients treated with peritoneal dialysis.

## 5. Fibrosis in Chronic PD Model

Our model of chronic peritoneal dialysis in rats also served for evaluation of the potential approaches which can result in better preservation of the peritoneum during chronic dialysis. We discovered that not only glucose but also other components in the standard bioincompatible solutions such as high content of GDP, low pH, and low content of lactate could be responsible for peritoneal fibrosis [38]. The daily use of glucose-based peritoneal dialysis solutions in a chronic peritoneal dialysis rat model was associated with morphological changes consistent with peritoneal fibrosis [38]. Whereas these changes in general were associated with the dialysate glucose concentration, the use of a more physiological bicarbonate/lactate-buffered peritoneal dialysis solution reduced but could not totally eliminate these effects [38]. We found that supplementation of the dialysis solution with precursors for glutathione synthesis in rats chronically exposed to peritoneal dialysis resulted in reduced fibrosis and neoangiogenesis within the peritoneum [63]. The protective effect of the glutathione supplemented solution in such conditions was additionally confirmed by* ex vivo* experiment studying the effects of dialysate effluents obtained from rats on the* in vitro* fate of cultured peritoneal mesothelial cells from the rats. We found that effluents from animals treated with glutathione caused weaker stimulation of the* in vitro* collagen synthesis compared to the control group [63]. Results from that study confirm that addition of the* ex vivo* testing of the dialysate effluents on the* in vitro* cultured cells helps in understanding the* in vivo* pathomechanisms. In another study, we documented that supplementation of the dialysis fluid with an ACE inhibitor, enalapril, inhibits peritoneal fibrosis in chronically dialyzed rats which resulted in better net UF at the end of the 4-week study [43]. The TGF/Smad pathway appeared to play a role in this process, and we hypothesize that high-glucose peritoneal dialysis solutions, particularly bioincompatible peritoneal dialysis solutions, activate this pathway which may contribute to the observed changes in the peritoneum [38]. The addition of rosiglitazone to standard dialysis fluids can maintain the peritoneal morphology and increase ultrafiltration in a peritoneal dialysis rat model [40]. An intraperitoneal PPAR-*γ* agonist may ameliorate morphological and functional changes of the peritoneum induced by standard peritoneal dialysis solutions in a chronic peritoneal dialysis rat model, while PPAR-*γ* agonists in rats treated with newer biocompatible solutions showed less benefit [40]. Furthermore, adenovirus-mediated gene transfer of active transforming growth factor (TGF-*β*) into the peritoneum is also a useful technique in inducing peritoneal fibrosis similar to that observed in patients undergoing long-term peritoneal dialysis [7–17].

## 6. Spontaneous Peritonitis in Rats Undergoing Chronic PD

The use of bicarbonate/lactate mixture and/or bicarbonate results in improvements in various biocompatibility measurements as compared with acidic, lactate-buffered solutions [30, 64–69] although this was not clearly demonstrated in all* in vivo* studies [70, 71]. In our next study, all peritoneal dialysis related procedures such as infusion and drainage were performed in a semisterile setting to induce spontaneous peritonitis [37]. Each day, dialysis fluid was infused in the morning (20 mL) and was drained after a 4-hour dwell [37]. Then the animals were reinfused with 20 mL of fresh solution. Samples for culture were collected once per week (4 hrs dialysate effluent) or when peritonitis was suspected (based on the peritoneal cells count (PCC) and/or effluent macroscopic evaluation) [37]. Upon the diagnosis of peritonitis, rats were treated by peritoneal dialysis for five or more days (if at all possible) and sacrificed by overdose of anesthesia [37]. We scored peritoneal adhesions in all animals at the end of the experimental study. Spontaneous peritonitis in rats on chronic PD, which could be diagnosed by PCC dialysate (PCC more than 3,000/mm^3^ or a 3-fold increase of PCC), occurs as a linear function of time, allowing this model to be used for evaluating the susceptibility of infection (peritonitis) and the inflammatory response following intraperitoneal use of different solutions [37]. Bicarbonate/lactate-buffered solutions reduced the time to infection, improved (enhanced) the inflammatory response, and reduced the adhesion formation in the peritoneal cavity [37]. This indicates that, in addition to the high-glucose concentration, other toxic factor(s) in the standard glucose lactate-based peritoneal dialysis fluid, such as lower pH, higher GDP content, and lactate buffer, may contribute to more severe peritonitis and, subsequently, increased adhesion formation [37]. These data are in broad agreement both with our previous chronic* in vivo* studies, which showed that the use of antibiotics was associated with low incidence of intraperitoneal infection and a low rate of catheter obstruction and excellent technique survival [27, 30].

## 7. Effect of Peritoneal Dialysis on the Renal Function and Morphology

Preservation of the renal residual function is an important factor determining adequacy of dialysis. Survival of patients treated with chronic peritoneal dialysis is better in a group with preserved renal function [72]. We tested the effect of chronic peritoneal dialysis in rats after unilateral nephrectomy treated for 12 weeks with peritoneal dialysis on morphology and function of the remaining kidney [42]. Animals were infused twice daily with 20 mL of hypertonic dialysis fluid, allowing it to absorb gradually from the peritoneal cavity. Rats with a removed kidney but not treated with peritoneal dialysis were used as control. Although there was no difference in renal creatinine clearance between the studied groups, at the end of the experiment urinary albumin excretion was four times higher in rats treated with peritoneal dialysis and urinary excretion of N-acetyl-*β*-D-glucosaminidase increased as well (+28%, *P* < 0.01). Glomeruli in the remaining kidneys were equally hypertrophied in both groups. However, in rats treated with peritoneal dialysis, the amount of PAS-positive substances in the glomeruli and the amount of collagen in the peritubular area were higher than in the control group by 69%, *P* < 0.001, and 274% *P* < 0.001, respectively. Results of this study show that our animal model of chronic peritoneal dialysis is suitable for studying the effect of such treatment on morphology and function of other organs. In another study, we found that chronic peritoneal dialysis in rats causes not only fibrosis of the peritoneal membrane, but also stimulates growth of the connective tissue within the liver [45]. In all animals, folding of the surface of the liver parenchyma was found to be due to penetration of the connective tissue elements between the hepatocytes. Due to such changes, groups of hepatocytes became detached and isolated from the remaining cells of the liver lobules. 

## 8. Rat Model of Chronic Uremia on PD

Studying the effect of uremia on both peritoneal transport and biocompatibility of dialysis solutions is generally recommended; however, such experiments are laborious, difficult, and expensive, and only a few papers report on peritoneal dialysis in uremic animals. Gotloib et al. described a model of peritoneal dialysis in rabbits made uremic by partial nephrectomy (total nephrectomy on one side and 5/6 nephrectomy on the opposite side) [73]. However, in some of these models, the level of uremia was moderate [1, 6]. Furthermore, no detailed description of how uremia influences peritoneal permeability has been provided. In our study, we performed a bilateral total nephrectomy [58]. We evaluated the effect of uremia on peritoneal permeability in anephric rats [58]. Bilateral nephrectomy caused acute uremia in the studied animals. Removal of just one kidney induced no significant changes in blood urea or creatinine levels [58]. However, already at 36 hours after removal of the second kidney, blood creatinine increased from 0.54 ± 0.09 mg/dL to 9.47 ± 1.06 mg/dL (*P* < 0.001) and blood urea increased from 34.5 ± 6.0 mg/dL to 465.6 ± 50.8 mg/dL (*P* < 0.001) [58], similar to what is observed in patients without renal function and treated with CAPD. The animals required intensive dialysis, on some days with an increased number of exchanges (4–6 exchanges per day). To obtain adequate ultrafiltration, exchanges with hypertonic dialysis solution were necessary. However, the transperitoneal equilibration of creatinine was faster in uremic animals [58]. Common symptoms experienced by the uremic animals included diarrhea and decreased appetite and resulted in loss of body weight [58]. This has also been observed in other studies [1, 73]. We think that systemic changes induced by uremia—such as overhydration, hyperosmolality, change in blood pressure, and decreased hematocrit—can also influence peritoneal permeability [58]. Monitoring these parameters in an animal model is very difficult.

The experimental model of uremia in anephric rats described here may also be appropriate for evaluation of new biocompatible dialysis solution. Testing these parameters in the same animal, before induction of uremia and after development of uremia, may reduce variations in the results owing to interindividual changes [58].

## 9. Mouse Model of PD

The transgenic mouse and cellular models have already made a significant impact on defining basic mechanisms that operate in the peritoneal membrane. The development of transgenic mice for describing pathways and molecules relevant to specific diseases together with the possibility of investigating minute biologic samples for numerous parameters simultaneously explains why the use of such models is set to transform research into practice [18]. To date, studies in null mice and cells derived from these animals provide direct mechanistic insights into the transport properties of the peritoneal membrane, the role of cytokines and chemokines in regulation of peritoneal inflammation, bacterial clearance and leukocyte recruitment, and pathways involved in structural and fibrogenic alterations that contribute to treatment failure [18]. Injection of IL-17 i.p. in experimental animals resulted in a time-dependent increase in the total number of cells in the peritoneal cavity [20]. Although IL-17A is generated by cells associated with adaptive immunity it appears to promote innate immune responses [19]. Our data indicate that the IL-17A-driven release of G-CSF from mesothelial cells may be an element of the peritoneal inflammatory response [19]. Mouse models also offer a vital preclinical resource in which the testing of various therapeutic strategies, arising from the mechanistic approaches, can be evaluated [18]. Barreto et al. found that pyrophosphate delivered via the intraperitoneal route using a PD solution does not appear to be deleterious to bone tissue in mice with chronic kidney disease [74]. This study indicates a potentially safe dose range which could be considered for future studies in the clinical setting [74]. Limitations of such models should be kept in mind, including the various growth and metabolic rates, the effect of the genetic background, and the possibility of adaptive mechanisms [18]. Despite these limitations, they nevertheless offer a tremendous resource that is poised to transform peritoneal research and lead to targeted interventions to prolong PD therapy [18].

## 10. Encapsulating Peritoneal Sclerosis (EPS)

EPS is a rare disease and the true incidence is unknown. It is not exclusive to PD but this review will focus on patients on PD or former PD patients [75]. For that reason, experimental models have been developed, which induced an EPS-like pattern by means of intraperitoneal application of several agents, for example, acidic glucose solution with pH inferior to 4 [76], chlorhexidine gluconate [77, 78], chlorhexidine acetate, povidone iodine, and also formaldehyde [79], as well as bleach plus whole blood [4]. Recently, mouse models have been established, which offer the possibility of investigating peritoneal transformations in the context of genetic alterations with specific regard to molecular mechanisms involved in that process [18]. Devuyst et al. investigated mice deficient in water channel aquaporin 1, which is involved in the transperitoneal transport or knock-in mice with mutants of gp130 involved in IL-6 signaling, which promotes the recruitment of T cells to the peritoneum [18]. Knowing that use of PD fluid containing GDPs generates advanced glycation end-products (AGE) [80], a comparison of wild-type mice with mice deficient in AGE receptor (RAGE) was performed. Peritoneal changes including inflammation, neoangiogenesis, and fibrosis can be mediated in a RAGE-dependent fashion [81].

## 11. Conclusions

If put into practice, using animal models as described in this review may improve our understanding of underlying problems in peritoneal dialysis using interventions that cannot ethically be applied to inpatients, and such studies could therefore potentially contribute to solving crucial clinical problems, thereby lengthening peritoneal dialysis patients' lives, as well as permitting a longer and safer application of this type of dialysis modality in individual patients [3, 27, 30, 37, 58]. Recent advancements in virology have led to the development of a potent, safe, and nonpathogenic adenoassociated virus [82]. Similarly, innovations in nanotechnology have rendered numerous efficient and safe nanoparticles for gene therapy [82]. This study shows that both gold nanoparticles and adenoassociated virus mediated decorin gene therapies significantly decrease peritoneal fibrosis* in vivo* in a rodent model [82]. This approach has potential clinical translational in providing a therapeutic strategy to prevent peritoneal fibrosis in peritoneal dialysis patients [82]. Further studies conducted in animal models of peritoneal dialysis will allow the development and improvement of this important method of renal replacement therapy.
